# Sex work and modes of self-employment in the informal economy: diverse business practices and constraints to effective working

**DOI:** 10.1017/S1474746414000426

**Published:** 2015-01

**Authors:** Jane Pitcher

**Affiliations:** Department of Social Sciences, Loughborough University E-mail: j.pitcher@lboro.ac.uk

**Keywords:** Prostitution, sex work, labour rights, self-employment, policy

## Abstract

This article draws on research with adult sex workers in indoor settings in Great Britain to explore diverse forms of self-employment, employment relationships and small business development, set within the context of changes to the wider economy. It considers how external constraints such as the legal context, social stigma and dominant policy discourses can impact on sex workers’ autonomy and actively work against their safety and wellbeing. The article argues that broad policy and legal approaches which fail to recognise the complexity of sex work constrain sex workers’ opportunities for business development and improvement of their working circumstances. It suggests the need for recognition of sex work as legitimate labour, as a prerequisite for policy changes to support sex workers and pave the way for improved working conditions, not only in managed settings but also facilitating collective arrangements and independent lone working.

## Introduction

Sex work remains a highly contested topic in policy debates. It has been argued that an ideology equating prostitution with gendered exploitation has influenced twenty-first century policy formation in the USA, the UK and parts of Europe (Weitzer, 2010). There are repeated pressures in the UK and Europe to adopt the ‘Swedish’ model, which criminalises the purchase but not the sale of sex. However, this model has been criticised on many grounds, particularly because it appears to have resulted in a more dangerous and stigmatised working environment for sex workers (Levy and Jakobsson, 2014). The failure of policy discourses to recognise sex work as legitimate work, and to include sex workers in discussions, impedes development of initiatives to improve their labour rights and working conditions. Recognition of sex work as a form of labour, however, does not preclude considerations of exploitation or interrelated issues such as labour market segregation and relative power and disadvantage. Moreover, viewing sex work through this lens can inform policy, through highlighting diverse experiences within the industry, drawing parallels with other forms of work and identifying ways to facilitate safer and more supportive working environments.

Much academic inquiry in recent years has turned to organisational structures and labour processes in sex work. Because of the semi-criminalised nature of sex work in the UK, sex markets tend to fall within the informal and unregulated economy, although they also intersect with mainstream industries and support services (West and Austrin, 2002; Sanders, 2008). It has been argued that the contemporary sex industry is comparable to other sectors in the formal economy, in that it displays variety in occupational structures, requires a range of skills and is influenced by local labour markets and global economic forces (Brewis and Linstead, 2000). Broader labour market changes of relevance to this article include the increase in non-standard employment relations, for instance the use of casual workers, who lack the job security of direct employees (McDowell, 2009). Non-standard arrangements include ‘dependent’ self-employment, where self-employed workers are in a subordinate relationship with a third party, without the autonomy of independent self-employed contractors who manage their own labour (Muehlberger and Bertolini, 2008). The constraints and opportunities influencing people's entry into jobs in the broader economy, which include reduced labour market options, increased demand for personal services, and the need for flexible working practices to accommodate women's domestic responsibilities, are similar to those shaping the entry of many women and migrant workers into the sex industry (Agustín, 2003; Bruckert and Parent, 2006). While lack of alternatives and economic pressures are often seen as motivations for engagement in sex work, it is important to recognise that sex work may also be actively chosen, particularly when considering independent or collective arrangements, which have been given less attention in policy debates than other areas of work in the industry.

This article draws on a study with adult sex workers in Great Britain to explore varied types of self-employment, working conditions and autonomy in different indoor settings. To set this in context, I begin by outlining the legal and policy framework relating to indoor work, specifically direct sex work,^1^ which is regulated differently from sectors within the wider sex industry such as erotic dance (Cruz, 2013). I argue that policy approaches which fail to recognise the diverse nature of sex work not only constrain sex workers’ attempts to make a living, but actively work against their safety and wellbeing and compromise their agency. I discuss the need for policy initiatives which facilitate sex workers’ rights and improve working conditions in managed, independent and collective settings.

## The UK legislative and policy context

National policy in the United Kingdom has tended to be enacted through legislative measures to control elements of commercial sex and mechanisms to rehabilitate sex workers (Scoular and Sanders, 2010). Prostitution policy is often located within ‘Violence against Women’ strategies nationally and locally (for example, Crown Prosecution Service, n.d.). This allows no space for the inclusion of male or transgender sex workers, who may also have specific service needs (Whowell and Gaffney, 2009). Policy documents such as the *Coordinated Prostitution Strategy* (Home Office, 2006) have tended to present a picture of sex work that does not reflect diversity within indoor settings and fails to acknowledge the possibility of voluntary commercial sexual exchange (Sanders, 2009). Policy approaches are also complicated by the fact that UK legislation makes no distinction between types of premises, working conditions or arrangements. Workers’ collectives, large managed parlours/saunas and smaller working flats may all be encompassed under the definition of ‘brothel’. Differences in management styles, degrees of professionalism and types of business ownership, from larger corporations to small businesses, are not taken into account.

As a result of inconsistent laws, sex workers are faced with a contradictory situation where, although it is not illegal for one consenting adult to sell sex to another, a number of measures constrain their working circumstances. Changes to the law in recent years have made conditions increasingly precarious for sex workers who work together or in rotation from the same premises. The Sexual Offences Act 2003 (Great Britain, 2003) extended definitions regarding brothel management and introduced new clauses relating to inciting or controlling prostitution for gain. Other legislation includes the Proceeds of Crime Act 2002 (Great Britain, 2002), which allows for assets to be seized if individuals are deemed to be keeping or letting premises for use as a brothel or living on the earnings of another's prostitution. While policy guidance from the Association of Chief Police Officers (2011) places emphasis on safeguarding sex workers, the law is applied inconsistently across the UK. In some areas there is evidence that the laws relating to brothel-keeping or controlling for gain are being used against receptionists and women working with others for safety (Carline, 2011; English Collective of Prostitutes, 2011). This can also result in sex workers being reluctant to report violence against them, for fear of the potential repercussions for themselves or others (Sanders *et al.*, 2009).

As Nussbaum (1998) and others have argued, removing the illegal status from adult sex work may help protect sex workers’ human rights and reduce the harms perpetrated against them. The preferred approach for many sex worker activists is decriminalisation^2^ of adult sex work, which in principle allows it to be regulated along the same lines as other industries (Koken, 2010). Abel and Fitzgerald (2010) demonstrate that decriminalisation has increased the rights of sex workers in New Zealand, although it has not reduced stigma, nor addressed all structural inequalities. As Day (2008) observes, state recognition of sexual labour as work does not necessarily result in greater autonomy for sex workers. Inequalities in the workplace are not unique to sex work, however, and as with other occupations, it is important to consider ways to monitor compliance with employment legislation and address unfair practices (Mossman, 2010).

### About the research

This article draws on a study of female, male and transgender adult sex workers in indoor settings in Great Britain. Semi-structured interviews were undertaken during 2011−13 with thirty-six current sex workers, and two receptionists and two managers in parlours^3^ who were former sex workers. Fifteen female participants worked as independent or agency escorts and nine female sex workers were based in managed premises, whereas the nine male and three transgender participants worked independently. Interviews explored participants’ working experiences and background, and perceptions of the terms, conditions and nature of their work. Recruitment was through online networks, escort websites, services for sex workers and snowballing. While it was possible to make some comparisons between the experiences of female and male sex workers, the number of transgender participants was too small for comparative analysis.

## Employment relationships and self-employment in indoor settings

The UK sex industry is characterised by non-standard working arrangements, particularly self-employment (Cruz, 2013). My research found variations in modes of self-employment comparable to those in the broader economy. The position of independent sex workers was similar to that of self-employed lone traders in other labour market sectors. They set their own terms and conditions, determined the services they would offer, their working hours and rates of pay. As Rachel, an independent worker in a collective, commented, independent sex work was ‘essentially like running your own business’, with both the flexibility and the financial uncertainties associated with self-employed work generally. Brothel and agency workers, however, while having notional self-employed status, were at the same time in an employment relationship with a third party, to whom they handed over a proportion of their wages. Agencies hiring sex workers took on responsibilities such as marketing and client liaison, although workers were usually able to negotiate their rates, working hours and services. Within brothels/parlours, the management stipulated conditions and hours of work, rates of pay and the services offered by the establishment. Although some degree of flexibility was accorded to individual workers, for example in terms of the services they provided, they were nonetheless restricted by the management rules. In this sense, they were similar to dependent self-employed workers in other sectors, although the illegal status of managed premises for commercial sex in the UK increased the precariousness of their circumstances. Sasha, a worker in a managed flat, observed that if problems did arise, workers had no formally recognised mechanisms for asserting their rights, either in relation to managers or clients: ‘because we’re outside the law, we’re vulnerable’.

### Work organisation in managed settings

As previous research has found (Pitcher, 2010), while there is a sizeable proportion of female sex workers based in brothels/parlours, male and transgender sex workers are more likely to work independently than in managed settings. Male participants in the current study commented that there were relatively few employment opportunities for male sex workers in brothels or agencies; only two had worked in managed premises, subsequently moving into independent sex work; and the transgender participants had only worked independently. Lack of alternatives and structural constraints are often associated with women's work in the managed sector (Sanders *et al.*, 2009). The long and irregular hours involved in independent self-employment or agency escorting sometimes made these less viable options for female participants with young children. For example, Louise stated that she had to move from escorting to parlour work when she had her first child. Nonetheless, some had actively chosen this form of work over self-managed options because they preferred to be in a job with set hours where a third party took on administrative tasks. Working in a more structured environment could also enable a clearer demarcation between work and private life, which was important for those who hid their working life from family and friends, and was also seen by some as beneficial for their work-life balance:

I’ve not particularly wanted to work as an independent, because I don't want all of those claims on my time, having to keep up with the chat boards on Punternet and all this stuff. I want to go to work, work, finish, come home. (Sasha, worker in managed flat)

While brothel work does not command the same hourly rates as independent or agency sex work, it was viewed by participants as offering better financial rewards than many service sector jobs, despite the amount handed over to third parties such as managers or receptionists. Although the shifts were often long, they could earn more over two to three days per week than working full-time in comparable jobs in the formal economy. Participants in managed settings also compared other aspects of their current work favourably with previous jobs outside the sector. Rebecca saw her current working situation in a small managed flat as involving greater flexibility and a degree of co-dependency. Cleo, an independent worker who had previously worked in parlours, noted that in comparison with employment in other sectors:

even if you’re in a situation where you’re working set hours, at set costs, you’ve got a lot more say in a way about your job . . . than in the kinds of work I was previously used to.

For participants such as Rebecca and Cleo, the relative autonomy over their work processes was an important feature of their job. Nonetheless, as Bruckert and Parent (2006) have observed, the intimacy of the labour and lack of guidance on the rules of engagement in commercial premises may make sex work more emotionally demanding than other service sector occupations. Several participants commented on the need to be ‘strong-minded’ when negotiating, and sometimes needing to enforce boundaries with clients or manage their emotional expectations. Participants had received little training on dealing with challenging situations and most of their learning was obtained on the job.

While participants noted that management policies in their current premises were aimed at providing a safe and supportive working environment, they had sometimes encountered less favourable working conditions or, in some instances, exploitative practices. Management practices appeared to be equally variable in male establishments. Cleo commented that in many instances managers of agencies or brothels had ‘not necessarily had much experience in other areas of work. So they kind of make it up as they go along.’ Because brothel management is illegal in the UK, although some premises operate certain good practice principles, the development of consistent formal workplace standards across the sector is inhibited (Sanders, 2009; Whowell and Gaffney, 2009). While it would be unrealistic to expect that legal changes alone would eliminate poor or exploitative practices, bringing sex businesses into the formal economy could increase the potential for implementation of standards for training, occupational health and safety, and collective organising, along the same lines as for other businesses.

### Independent work and business practices

Independent participants in the study viewed themselves as exercising greater autonomy and control over their working conditions than those in an employment relationship in either the sex industry or mainstream service sector occupations. For participants such as Ruby, self-managed sex work was:

a career choice, it's a business choice. I don't do anything I don't want to do. I set my hours, I set my limits, I set my prices. The independence is vital to me.

A significant development influencing labour processes in independent sex work has been the role of the internet (Bernstein, 2007; Walby, 2012). As participants observed, the internet was important because it enabled them to establish an online profile with relatively minimal effort or start-up costs, and facilitated networking and communication with clients. This opened up opportunities for those with few qualifications to move from low-skilled and poorly remunerated jobs with limited responsibility into managing their own business. For example, Jessica's previous employment had been: ‘everything you would expect a woman with no qualifications to have done . . . shop assistant, cleaner, barmaid . . . I was a crab dresser on the fish market . . .’. In comparison, as a self-employed escort, she ran her own enterprise and had developed a range of skills relating to financial management and business organisation, as well as design and management of her marketing website, and commented that ‘I love every minute of it most of the time’.

The diversity of skills required in small business management contributed to participants’ job satisfaction, although, as with any business, there were moments of tedium and the risks associated with lone working could sometimes make the work stressful. Jodie, an independent escort, noted that: ‘you need to . . . have the awareness of what you’re doing for physical safety, health safety-wise’. Some participants had prior experience of business management which helped to inform their commercial strategies. Others drew on the knowledge of other sex workers or subcontracted certain aspects of their business, for example, employing web designers or accountants. Some participants had encountered situations where third parties had tried to exploit them financially or sexually, however, and as Carla remarked, ‘you have to be careful’ when recruiting external service providers to ensure they respect professional boundaries.

While participants were often cautious about divulging their occupation to third parties, some independent workers were more open about their status. More than half of male participants, one transgender and four female sex workers, stated that not only were their friends and family aware of their working circumstances, but sometimes others outside their immediate social circle, including service providers such as financial advisors, or colleagues on training courses. Jessica commented:

if you behave as if you’re doing something wrong then you’re basically saying to people ‘yes I should be ashamed of this’ . . . if you act like you’re just doing something perfectly ordinary most people treat you like you’re doing something perfectly ordinary.

Although such openness might help to normalise sex work as a legitimate labour market activity and challenge social stigma, for many the risk of discrimination was too great an obstacle for disclosure of their work identity. The majority of participants, particularly female workers, kept their working life secret, which could contribute to their social isolation.

Sex work is often seen as a temporary arrangement for instrumental purposes, although for some participants it represented a longer-term occupational pathway. Individual trajectories related to factors such as participants’ aspirations, relative job satisfaction and career development opportunities. Some participants were content to remain as self-employed sole operators, which gave them a satisfactory income, autonomy and time to pursue other interests. Some had plans to develop their sex work enterprise, sometimes branching out into new markets. For example, Jemma and Martin had financed their studies through sex work, and used the knowledge from their training to develop their businesses in order to provide more holistic sexual services, which intersected with mainstream therapeutic occupations. Others had diversified into new spheres within the sex industry while continuing with sex work: for example Jake had built advertising websites associated with the sex industry; Jessica and Leon became involved in developing peer support for other sex workers. Sometimes participants undertook sex work alongside an occupation in the formal economy. Having an alternative career could help to mitigate the uncertainties of self-employed sex work, including, as Verity observed, the impact of changes to the law which might cause business to go ‘really, really quiet’ at times.

Although some independent participants preferred to work alone, for others collective working offered the potential for safer working arrangements, companionship and the ability to share business-related costs. Styles of collective working included participants working independently and sharing costs equally; one person renting premises, with others using the property contributing to the costs; and situations where one worker took on an administrative role and charged a nominal fee for rent and other costs, which blurred the lines between collective and managed work. While collective working could address some of the disadvantages of lone working, however, there were implications not only in relation to the laws on brothel-keeping and controlling for gain discussed earlier, but also in terms of potential breach of tenancy agreements.

## Constraints to business practices and views on policy

A key factor differentiating sex work from mainstream occupations is the way state strategies limit sex workers’ autonomy through constraining their ability to develop their own business, for example, through working with other sex workers or bringing support services in house. As Christopher, an independent escort, commented, fear of the possible legal consequences made it ‘very difficult to do the job as you’d want to do it’. Jodie observed that:

if I want to open a shop, or any sort of service, I can employ a load of employees. You can't do that with this job. Indeed, I can't even work in a syndicate.

Thus, more unstable forms of business transaction are imposed upon the sector. For many independent participants, the potential legal repercussions acted as a strong deterrent to collective working. Carla expressed her frustration at the inconsistencies in the law, whereby she could be legitimately self-employed, yet prohibited from working with others:

It makes me angry that I pay tax, yet if I was to want to work with somebody else in another house for safety, or share a house with somebody to keep my costs down, it's illegal.

Participants across collective and managed settings voiced concerns about their precarious legal status and lack of support for safer working practices. The possibility of prosecution or having their assets seized if they were deemed to be managing others was a key concern. For those who had worked in the unregulated economy for most of their lives, any savings put by from their work could represent retirement income and thus there were significant risks for mature workers. For example, Carol had moved from selling sex to managing a small flat, where she worked with friends. While the local police had previously been tolerant of establishments such as hers, where there was no evidence of exploitative practices, in recent months they had started to take a more punitive approach towards all managed premises and she was facing prosecution. She risked losing the savings she had built up and also her home.

Participants expressed concern that their voices continued to be excluded from policy debates, which were often seen as reacting to political campaigns, leading to the perpetuation of misrepresentations about their industry and exacerbating their precarious work situation:

it's almost like a forcing of statistics and opinion on people that everyone that does this is a victim and vulnerable. I’m sorry but that's completely false. And actually the law makes us vulnerable. And victims. (Rachel, worker in collective)

Many participants argued that their work should not be criminalised, and wanted to see policy changes to improve their safety and welfare, although there was no clear consensus regarding the shape this should take. Treating sex work as a viable form of labour was seen as a starting point for change and the right to equal treatment:

we’d have better working conditions if we were not criminalised and if we were recognised as workers with rights . . . the worst thing with stigma is that . . . people think you’re a victim or that you’re alienated [and] that your opinion doesn't matter . . . it's like denying your intelligence . . . and I think viewing you like a child. (Pascal, independent sex worker)

Participants who were aware of campaigns to criminalise the purchase of sex saw this as impinging even further on their rights as workers, as it would take away their livelihood, did not reflect the positive relationships they had with many clients and would exacerbate their social exclusion. As Sasha commented, the consequence would be that the industry would become ‘a much nastier, underground thing’. This would have implications for workers in all settings, including those in the independent sector, as it would delegitimise consensual transactions between individuals. Workers would find it problematic to be transparent about their status, as this could pose a threat to their clients and thus the independent sector would become even more hidden.

## Conclusions

Modes of self-employment in the indoor sex industry, from dependent workers to independent entrepreneurs, are similar to those in the formal economy. Nonetheless, the fact that the only legitimate business arrangement is currently self-employed lone working brings into question the role played by the state in determining working relationships in the sex industry. Despite the diversity of adult sex work, no distinction is made in law or policy between good or poor management practices, or between supportive or exploitative environments. Criminalisation allows exploitation of workers to go unchecked (Sanders, 2009), while the current legal and policy framework prohibits recognition of sex work as a form of labour and the development of professional standards.

While decriminalisation may be an essential prerequisite for recognition of sex workers’ rights to the same employment protections as other workers, it is not sufficient to eradicate poor management practices (Mossman, 2010). As Brents and Hausbeck (2010) have observed, in states where the sex industry has been mainstreamed it continues to reflect wider labour market inequalities. Moreover, not everyone in the sex industry would welcome incorporation of sex work within the formal economy, with the potential implications for taxation and standardisation of working practices, which could impact not only on current flexible working arrangements but also on employers’ profitability and sex workers’ earnings. Decriminalisation would remove some of the barriers to establishment of collective enterprises, as sex workers should be afforded the same freedoms as other workers to develop small businesses. Nonetheless, there are key players in the sex industry who influence its regulation (Scoular and Sanders, 2010), and this raises the question of whether a mainstreamed industry might favour the interests of larger business operators over those of smaller cooperatives.

Recognition of sex work as a legitimate form of labour may be seen as a starting point for acknowledging sex workers’ entitlement to workplace rights. It is not possible here to explore detailed mechanisms through which those rights might be realised, particularly as interests vary according to the working context, with the needs of independent workers being very different from those of workers in managed establishments. This study indicates that addressing exploitation or poor management practices in brothels is important for male as well as female sex workers, although given the predominance of managed premises employing women, the impact of any policy changes would be greatest for female sex workers. Labour rights and protections in managed premises should, in principle, provide a basis for addressing variable conditions and inequalities, for sex workers and those in supporting roles. For independent workers, formal recognition of their occupation might enable the development of professional standards and guidance, which would be useful not only for new entrants to independent sex work, but also for establishing codes of conduct for contractual relationships with third parties. While female participants appeared to be more likely than men to conceal their working identity, the study did not otherwise identify distinctly gendered patterns in independent participants’ approaches to business practices. Nonetheless, further research may be required to explore whether there are gender-specific issues which policies need to consider. Much could be learned from the experience of countries which have undertaken regulatory reforms to support sex workers’ rights, particularly through decriminalisation, although the structure and size of the sex industry in these countries may differ from that in the UK. In the same way that the voices of other workers are encouraged in social dialogue, it is important that sex workers from different sectors in the UK industry are central to debates on the future of their industry, to ensure that policy formation is built upon their knowledge and experience of diverse working practices, rather than shaped on the basis of moral campaigns.

## Notes

1 1 Direct sex work involves the sale of sexual services, for example through escorting or in brothels.

2 Decriminalisation removes the criminal penalties from adult sex work and regulates it in the same way as other forms of work, with sex workers entitled to the same labour rights as workers in other sectors.

3 Parlours/saunas are alternative terms for brothels in the UK.
